# Putative *Otobius megnini*-associated clinical signs in horses in South Africa (2012–2018)

**DOI:** 10.4102/jsava.v91i0.1983

**Published:** 2020-07-07

**Authors:** Sean M. Miller

**Affiliations:** 1 Summerveld Equine Hospital, Durban, South Africa

**Keywords:** equine, tick, *Otobius megnini*, veterinary, parasitology, acarology

## Abstract

*Otobius megnini* has been associated with certain clinical conditions in horses in both California and Mexico. A number of cases similar to those described previously have been identified by the author in South Africa. This case report summarises these cases to demonstrate that the clinical condition occurs readily in South Africa and may be increasing in occurrence. The disease has minimal coverage in the literature making it more likely that a veterinarian, unfamiliar with the disease, will miss the diagnosis. The author would like to make veterinarians aware of this as a potential differential diagnosis. This study is a retrospective review of clinical data. Clinical records of patients with similar clinical signs and treatment were reviewed and grouped together as relevant cases for this case report. Ten cases of *O. megnini* associated neuromuscular dysfunction are reported, suggesting a link between the occurrence of the tick and the clinical condition. Clinical signs include third eyelid prolapse, localised muscle fasciculations, elevated heart rate and limb stamping. Serum chemistry changes commonly show increased aspartate aminotransferase and creatine kinase enzymes activities. The occurrence of the ticks within South Africa and the increasing number of cases presented demonstrate the need for more investigation into the pathophysiology of this condition.

## Introduction

*Otobius megnini*-associated disease in equines has been reported by Madigan et al., showing an association of the tick and clinical disease in five horses in 1995 in California (Madigan et al. 1995:74–76), whilst Zarate-Ramos et al. (2014) described a similar case attributed to *O. megnini* in Mexico in 2014 (Zarate-Ramos et al. 2014:16–20). As yet, no neurotoxin has been described for this tick and it is possible that there may be neuromuscular dysfunction (Aleman 2011:481–506) alone or in relation to neurotoxin involvement (Pecina 2012:531–532). *Otobius megnini* is not known to transmit any pathogens (Barker & Walker 2014).

*Otobius megnini* has a single host life cycle (Nava, Mangold & Guglielmone 2009:1–5) and is parasitic for long time periods in the ear of the host (Jongejan & Uilenberg 2004:S3–S14). *Otobius megnini* has a broad host range with inflammation, tissue necrosis and secondary bacterial infection the most commonly reported sequelae of infestation (Harris 1996:272–276). *Otobius megnini* occurs extensively throughout South Africa and can be easily transported by hosts to new locations (Nava et al. 2009:1–5; Walker et al. 2003), showing some seasonal infestation trends towards the warmer months (Nava et al. 2009:1–5). *Otobius megnini* undergoes multiple nymphal stages, whilst the adults are non-parasitic (Jongejan & Uilenberg 2004:S3–S14). Nymphs and larvae can remain on the host for approximately 3 months (Broom 1920:362–363; Jongejan & Uilenberg 2004:S3–S14). Humans and horses (Walker et al. 2003) have been reported as hosts as early as 1920. Suspected paralysis of a human patient associated with *O. megnini* was reported in 1958 in South Africa (Peacock 1958:201–202). In addition, a young girl suffering from *O. megnini*-associated otitis externa was reported in South Africa in 2001 (Naudé et al. 2001:118–119). In India, there are reports of the tick causing painful human ear infestations (Chellappa 1973:655–658). A report in Canada of euthanasia of horses because of ‘demented horses’ had necropsy findings that revealed large numbers of *O. megnini* in the ear canals with associated necrosis of the auricular and adjoining nerves. These findings implied an association of *O. megnini* with neurologic disease (Rich 1957:415–418). To the author’s knowledge there have been no subsequent descriptions in South Africa of similarly affected equines.

In a number of hosts, myopathy or paralysis has been associated with various tick species. Tick paralysis caused by *Ixodes holocyclus* is well documented in Australia, typically showing progressive lower motor neuron signs, weakness, ataxia and recumbency (Johnson 2008; Tee & Feary 2012:181–185), causing rapid ascending paralysis (Bootes 1962:68–69) and killing foals (Bootes 1962:68–69). With *I. holocyclus*, a salivary toxin is implicated (Grattan-Smith et al. 1997:1975–1987) with envenomation most common in small, young equids. Death is more common in the younger and smaller animal (Ruppin et al. 2012:175–180). The first step in all treatments is identifying and removing the offending ticks (Grattan-Smith et al. 1997:1975–1987) as removal generally results in recovery of paralysed animals. The use of acetylpromazine to act as a calming agent and minimise muscle activity is beneficial (Tee & Feary 2012:181–185). Treatment of *I. holocyclus* paralysis consists of tick antiserum and supportive care (Tee & Feary 2012:181–185). In North America, a neurotoxin secreted by engorged female *Dermacentor andersoni* or *Dermacentor variabilis* (Krishnan et al. 2009:358–362) causes similar signs.

A further differential diagnosis for this clinical condition includes myotonia where abnormal muscle contractions are seen, particularly in response to a stimulus (Cassart, Coignoul & Desmecht 2008:1–16). For example, congenital myotonia has been described where skeletal muscles have sustained contractions (Jamison et al. 1987:353–358). Toxic myopathies (monensin poisoning) may also display muscle tremors, ‘colic’ signs, tachycardia, tachypnoea with increases in serum levels of aspartate aminotransferase (AST) and creatine kinase (CK) enzyme activity (Harris 1996:272–276), whilst seasonal pasture myopathy affects respiratory and scapular muscles and is caused by ingestion of a toxin (Finno et al. 2006:1134–1141). Exertional rhabdomyolysis may also present with muscle tremors, tachycardia, tachypnoea and increases in serum levels of AST and CK enzyme activity.

A number of cases, similar to those described by Madigan (1995:74–76) and Zarate-Ramos et al. (2014:16–20), have been identified by the author in KwaZulu-Natal, South Africa. This case report summarises these cases to demonstrate that this is a clinical condition that occurs readily in this region. The increase in prevalence would indicate a need for more investigation into the pathophysiology of this condition.

## Materials and methods

Clinical records of patients with similar presenting clinical signs and treatment were reviewed. Cases were excluded if they did not have serum chemistry data, positive identification of the presence of *O. megnini*, third eyelid prolapse and muscle fasciculations. In all cases except one, blood samples were taken at the time of first clinical examination. All selected cases were Thoroughbred racehorses in the pre-training phase of their career that had recently arrived at one of two race training centres in KwaZulu-Natal, South Africa. Presentation was generally 2 weeks after arriving at the race training centres, either from yearling sales or from pre-training farms. As such, all horses were only in light work. None of the histories related any of the signs to work, or immediately post-work. The cases were tabulated to compare similarities.

### Patient presentation, management and outcome

The clinical findings and treatments are reported in Table 1, and the haematological and serum chemistry findings are reported in Table 2. Most commonly affected individuals were 2-year-old (eight out of 10) fillies (nine out of 10) and horses that had recently moved yards. The most common presenting complaints were stamping of hooves, muscle fasciculations and third eyelid prolapse. Less commonly seen signs included yawning, head tilting, circling and recumbency. In all cases, *O. megnini* ticks were identified and removed from the ears. Occurrence of this disease seems to cluster around February to May and September to October with the greatest number of cases occurring in May (three out of 10) and September (three out of 10) during spring and autumn, which may be associated with warmer months in the Southern hemisphere.

**TABLE 1:** Clinical findings and treatment of the reported cases.

Case no.	Date	Gender	Age (years)	Presenting signs and history	*Otobius megnini*identified	Treatment	Duration/outcome
**1**	09 May 2012	Filly	2	Heart rate: 48 bpm, cramping of legs, stamping of feet, intermittent third eyelid prolapse and muscle fasciculations. Recently moved to racing yard	Multiple in both ears	Dexamethasone (Colvasone§) 0.08 mg/kg IV, butylscopolamine bromide (Buscopan compositum¶) and phenylbutazone (Pheynlbutazone†) 4.4 mg/kg IV	10 days
	10 May 2012			Heart rate: 40 bpm		Acepromazine (Neurotranq†) 0.04 mg/kg IV and doramectin (Dectomax‡) 0.18 mg/kg IM	Recovered
**2**	13 September 2013	Filly	2	Constant third eyelid prolapse on exam. Muscle fasciculations. Continuous yawning. Recently moved to racing yard	Multiple in both ears	Acepromazine (Neurotranq†) 0.04 mg/kg IV, ivermectin (Solution LA 3.5%††) 450 *µ*g/kg SC and phenylbutazone (Pheynlbutazone†) 4.4 mg/kg IV	2 Days
							Recovered
**3**	21 October 2014	Filly	2	Marked third eyelid prolapse bilaterally, triceps muscle fasciculations, stamping of both front hooves and heart rate 48 bpm. Recently moved to racing yard	Multiple in both ears	Acepromazine (Neurotranq†) 0.04 mg/kg IV and dexamethasone (Colvasone§) 0.08 mg/kg IV	3 days
							Recovered
**4**	17 September 2015	Filly	2	Constant third eyelid prolapse of the right eye. Stamping of hooves. Unsettled. Recently moved to racing yard	Multiple in both ears	Acepromazine (Neurotranq†) 0.04 mg/kg IV	1 day
							Recovered
**5**	07 March 2016	Filly	2	Heart rate 52 bpm, constant left eye third eyelid prolapse, generalised muscular fasciculations of trunk and thorax and recumbent in the stable. Recently moved to racing yard	Multiple in both ears	Acepromazine (Neurotranq†) 0.04 mg/kg IV and ketoprofen (Ketofen‡‡) 2.2 mg/kg IV	2 days
							Recovered
**6**	09 May 2016	Filly	2	Single limb stamping and chopping associated with muscle fasciculations on the ipsilateral lateral thorax Recently moved to racing yard	Multiple in both ears	Flunixin meglumine (Pyroflam§) 1.1 mg/kg IV	1 day
							Recovered
**7**	13 May 2016	Filly	2	Stamping of hooves, chopping with front limbs and intermittent third eyelid prolapse mainly affecting the right eye. Recently arrived in racing yard	Multiple in both ears	Ketoprofen (Ketofen‡‡) 2.2 mg/kg IV, acepromazine (Neurotranq†) 0.04 mg/kg IV and ivermectin (Solution LA 3.5%††) 450 *µ*g/kg SC	1day
							Recovered
**8**	05 February 2017	Colt	1.5	Unilateral lower abdominal wall fasciculations and occasional recumbency with seizure-like paddling of hooves. Recently moved to racing yard	Multiple in both ears	Flunixin meglumine (Pyroflam§) 1.1 mg/kg IV, butylscopolamine bromide (Buscopan compositum¶) 0.17 mg/kg IV, acepromazine (Neurotranq†) 0.04 mg/kg IV and doramectin (Dectomax‡) 0.18 mg/kg IM	1 day
							Recovered
**9**	02 April 2017	Filly	1.5	Constant left third eyelid prolapse and muscle fasciculations of the right flank. Recently moved to racing yard	Multiple in both ears	Flunixin meglumine (Pyroflam§) 1.1 mg/kg IV and dexamethasone (Colvasone§) 0.08 mg/kg IV	1 day
							Recovered
**10**	16 October 2018	Filly	2	Stamping of hooves, chopping front feet and muscle fasciculations of triceps and hindquarters. Intermittent recumbency. Mild third eyelid prolapse with noise stimulation	Multiple in both ears	Acepromazine (Neurotranq†) 0.04 mg/kg IV, flunixin meglumine (Pyroflam§) 1.1 mg/kg IV	3 days
							Recovered

bpm, beats per minute; IV, intravenous, IM, intramuscular; SC, sub cutaneous.

† , Virbac, Fort Worth Texas, United States;

‡ , Zoetis, Parsippany-Troy Hills, New Jersey, United States;

§ , Norbrook, Newry, Northern Ireland;

¶ , Boehringer Ingelheim, Ingelheim am Rhein, Germany;

†† , MSD Animal Health, Johannesburg, South Africa;

‡‡ , Merial, Johannesburg, South Africa

**TABLE 2:** Haematological and serum chemistry findings for each case reported.

Case no.	Date	Haematological and serum chemistry abnormalities									
		Red blood cells (6.80 x10^12^/L – 12.90 x10^12^/L)	Haematocrit (32% – 53%)	Haemoglobin (11.0 g/L – 19.0 g/L)	Mean corpuscular volume (37 fL – 59 fL)	Mean corpuscular haemoglobin (12.3 pg – 19.9 pg)	Mean corpuscular haemoglobin concentration (31.0 g/dL – 45 g/dL)	Red cell distribution width (17.00% – 25.4%)	White blood cells (5.40 × 10^9^/L – 14.30 × 10^9^/L)	Neutrophils 2.26 × 10^9^/L – 8.5 × 10^9^/L)	Lymphocytes (1.5 × 10^9^/L – 7.7 × 10^9^/L)
1	10 May 2012	9.76	37.8	13.5	38.8	13.8	35.7	23	11.24	**10.00**	**0.45**
	16 May 2012	10.85	41.2	15.0	37.9	13.8	36.4	22.7	7.82	5.96	**1.04**
	17 May 2012	-	-	-	-	-	-	-	-	-	-
	19 May 2012	-	-	-	-	-	-	-	-	-	-
2	13 Sept. 2013	8.83	37.7	14.2	42.6	16.1	37.8	21.4	8.84	5.26	2,76
3	21 October 2014	11.84	42.95	16.6	**36**	14.1	38.8	**25.6**	11.00	5.55	4.92
4	17 September 2015	9.89	38.10	13.7	39	13.8	35.8	24.9	9.29	6.49	1.91
5	07 March 2016	10.33	39.25	14.4	38	14.0	36.8	**25.7**	10.71	6.09	3.80
6	09 May 2016	8.61	34.81	12	40	14.0	34.5	24.2	6.94	5.08	**1.32**
7	13 May 2016	9.87	38.90	14.6	39	14.8	37.6	24.4	9.81	6.34	2.95
8	05 February 2017	10.32	**31.16**	12.8	**30**	12.4	41.0	**27.6**	11.74	6.88	4.21
9	02 Apr. 2017	11.42	34.89	15	**31**	13.1	43	**29.2**	11.12	6.25	4.1
10	16 Oct. 2018	7.86	34.30	12.1	43.6	15.4	35.3	21.9	11.58	**9.39**	**1.41**

Note: Abnormal values in bold type.

**Table:** 

Case no.	Date	Haematological and serum chemistry abnormalities											
		Monocytes (0.10 × 10^9^/L –1.00 × 10^9^/L)	Eosinophils (0.00 × 10^9^/L –1.00 × 10^9^/L)	Basophils (0.00 × 10^9^/L –0.03 × 10^9^/L)	Creatinine (71 µmol –194 µmol/L)	Blood urea nitrogen (3.6 mmol/L – 8.9 mmol/L)	Total protein (56 g/L – 79 g/L)	Albumin (19 g/L – 32 g/L)	Globulin (24 g/L – 47 g/L)	Aspartate aminotransferase (100 U/L – 600 U/L)	Gamma-glutamyl transferase (0 U/L – 87 U/L)	Total Bilirubin (0 U/L –60 U/L)	Creatine kinase (10 U/L – 350 U/L)
1	10 May 2012	0.54	0.23	0.02	118	4.5	**55**	28	27	**>600**	18	43	**>2035**
	16 May 2012	0.60	0.18	**0.04**	211	9.6	53	26	26	**2466**	12	32	**1838**
	17 May 2012	-	-	-	**262**	5.4	-	-	-	**2985**	-	-	**994**
	19 May 2012	-	-	-	273	8.0	-	-	-	**2376**	-	-	**829**
2	13 Sept. 2013	0.51	0.26	**0.05**	-	-	-	-	-	**934**	-	-	**1783**
3	21 October 2014	0.05	0.42	**0.07**	-	-	-	-	-	**1908**	-	-	**>2036**
4	17 September 2015	0.32	0.53	**0.04**	137	5.1	61	25	36	**1788**	29	29	**4005**
5	07 March 2016	0.55	0.24	0.03	-	-	-	-	-	**718**	-	-	**564**
6	09 May 2016	0.33	0.18	0.02	97	6.1	61	29	32	**1408**	23	5	**>2036**
7	13 May 2016	0.43	0.08	0.01	-	-	-	-	-	**1995**	-	-	**>4072**
8	05 February 2017	0.27	0.35	0.03	97	5.4	63	31	31	335	25	32	**659**
9	02 Apr. 2017	0.49	0.24	**0.04**	141	6.8	64	33	31	439	43	27	**515**
10	16 Oct. 2018	0.48	0.26	**0.04**	159	6.8	62	28	35	559	75	41	**2473**

Note: Abnormal values in bold type.

In seven out of 10 cases AST and 10 out of 10 cases CK levels were raised. In case 9, AST and CK were only measured 4 days after the clinical signs first presented. The AST for this case was in the normal range and the CK mildly elevated. It is therefore difficult to associate these levels with the clinical condition. In the other two cases with normal AST values, the CK levels were both high (cases 8 and 10). The assumption would be that sampling occurred at a more acute phase where CK level was elevated, but AST was yet to be elevated. Serum CK peaks after muscle injury at approximately 4 h – 6 h and has a half-life of 6 h – 10 h, whilst AST has a much slower peak of 24 h – 48 h and a half-life of between 2 and 10 days (Cardinet, Littrell & Freedland 1967:219–226; Valberg et al. 1993:11–16; Valentine 2003:250–252). The mean AST for these cases was 1423.92 U/L (standard deviation [SD] = 852.31; range 335–2985). The mean CK for these cases was 1833.77 U/L (SD = 1133.54; range 515–4072). Note that not all readings had an absolute measurement and where this is the case the highest value recorded was taken as the value for the estimation of mean.

Cases 1 and 10 had mild neutrophilia accompanied by a mild lymphopaenia (case 1 had a lymphopaenia without neutrophilia on a follow up haematology). Case 6 had a mild lymphopaenia without a neutrophilia. On the follow-up examination, case 1 also had a basophilia, and cases 2, 3, 4, 9 and 10 had a basophilia. These may be endogenous corticosteroid-related stress response leukograms. Alternatively, the neutrophilia may be because of physical damage of the ear caused by the ticks within the ear. Cases 3, 8 and 9 had mild decreased mean corpuscular volume (MCV), and cases 3, 5, 8 and 9 had mildly elevated red cell distribution width (RDW). The microcytosis may be an artefact caused by delayed processing of blood samples leading to cell swelling, or may be because of the younger animals having a physiological normally lower MCV. The increases in RDW without other haematological changes related to anaemia may be because of fragmentation of cells during collection or agglutination (Satué, Hernández & Muñoz 2012:573–596). Case 1 had mild low total protein on two examinations and elevated creatinine on three examinations, and elevated urea on one examination. Because of the nature of a retrospective study, full serum chemistry data were not available for cases 2, 3, 5 and 7. Because of the strong suspicion of the clinical condition at the time of treatment, full serum chemistry was not run; AST and CK were run to confirm that they were elevated. In the third and fourth follow-up clinical pathology of case 1, no haematology was performed and only previously elevated serum chemistry was re-evaluated. The mean time to recovery was 2.5 days (SD = 2.62).

These 10 cases were documented over a 6-year period. A number of similar cases occurred during this period but because of poor record keeping and clients’ lack of knowledge of the disease, a number of cases were treated without the author’s knowledge or without accurate enough records to verify the cases retrospectively.

Mechanical removal of ticks is essential to alleviate the signs in the author’s experience. Treatment with acetylpromazine (Neurotranq^1^) 0.04 mg/kg and flunixin meglumine (Pyroflam^2^) 1.1 mg/kg as well as an ivermectin-type parasiticide are the first choice of medical treatments that are effective in the author’s experience. The use of non-steroidal anti-inflammatories and acetylpromazine is sometimes required for several days after diagnosis to treat on-going signs.

## Limitations

The small number of cases, some of which did not have complete serum chemistry data, was a limitation of this study. The lack of consistent clinical signs is also another limiting factor.

## Discussion

The occurrence of this condition has, as far as the author is aware, not been previously described in South Africa. Whilst few cases have been documented, the occurrence of the condition warrants further investigation. Seasonal peaks in larval stages in warmer months seem to coincide with the incidence of cases in this study (Diyes & Rajakaruna 2016:170–175).

It is unlikely that the condition, although occurring mainly in fillies, is related to exertional rhabdomyolysis because none of the histories reported excessive work or immediately post-work. The horses in this study were all new to their yards and were not under excessive training loads according to their trainers. However, this cannot be entirely ruled out in this study: muscle biopsies were not taken but would be useful for future studies. Testing for other myopathies was not performed.

The exact pathophysiology of this condition is not clear. Other tick toxicoses frequently result in paralysis, but ticks may also cause other hypersensitivity and immunological reactions (Mans, Gothe & Neitz 2008) with approximately 69 tick species causing some form of paralysis worldwide (Mans et al. 2008). Paralysis caused by soft ticks is associated with the prolonged feeding pattern of larvae and nymphs (Mans et al. 2008). In most species causing paralysis, this occurs in the engorgement phase where numerous protein products are produced by the salivary glands of the tick which affect the nervous system (Mans et al. 2008). However, it is not reported that *O. megnini* produces any toxin/s which could cause the neuromuscular dysfunction seen in these cases or whether its location within the ears may cause inflammatory reactions that cause neurological signs. The signs associated with these cases are also not consistent with those of other known paralysis ticks (Madigan et al. 1995:74–76; Rich 1957:415–418). There are indications that increased motor unit activity could be the cause of the signs seen in these cases (Madigan et al. 1995:74–76; Mans, Gothe & Neitz 2004:S95–S111). There is also potential malfunctioning of ion channels that may play a role in the pathophysiology of the condition (Cassart et al. 2008:1–16). Aleman suggests that *O. megnini* causes a myotonia by altering neuromuscular transmission at the postsynaptic membrane of the neuromuscular junction (Aleman 2011:481–506). Whether the condition is a true paralysis or a myotonia remains to be shown. It is clear to the author that there is some neuromuscular dysfunction and myotonia is more likely to be occurring which suggests that ‘*O. megnini*-associated neuromuscular dysfunction’ may be a more appropriate description of the condition based on the current limited knowledge.

*Otobius megnini* is well established in the local racehorse training centres and seems to be present on a large number of equines in the population. The occurrence of the condition in young horses that are relatively new to the stable yard suggests that there may be an element of immune suppression that allows the condition to occur in the new, presumably stressed, individual horses. Alternatively, there may also be a level of immunity that develops against the condition as older horses in the same yard are exposed to the same ticks but do not seem to succumb to clinical disease. A lack of immunity in naive horses may also explain their increased susceptibility on arrival in a new environment. There is evidence that Thoroughbreds may be more susceptible than crossbreds to infestation and that this may be related to the development of immunity (Diyes & Rajakaruna 2017:164–176; Wikel 1996:1–22). In addition, well-groomed horses, with shaved ears seemed to be more susceptible to infestation (Diyes & Rajakaruna 2016:170–175), although regular cleaning of shaved ears may allow frequent identification and removal of ticks.

Veterinarians should remain vigilant for this condition and ensure horse owners are informed about tick removal (Pecina 2012:531–532) and appropriate application of locally available parasiticides (Drummond 1985:111–119) as part of a holistic approach to the control of tick infestations. The condition in this study has been shown to occur in South Africa and, whilst simple to treat, veterinarians not familiar with it may misdiagnose the signs and treat incorrectly resulting in unnecessary morbidity and mortality of animals.

Future study is needed to determine if there is a toxic or physical cause to the condition and the true prevalence of both the ticks and the condition within the racing Thoroughbred population of South Africa.

## Footnotes

1 Norbrook, Newry, Northern Ireland

2 Virbac, Fort Worth Texas, United States
